# Modern Sociology

**Published:** 1902-08-30

**Authors:** 


### MODERN SOCIOLOGY.

### HOUSEWIFERY TEACHING IN BELGIUM.

THE Belgian Government having come to the conclusion that the girls who become the wives of working men receive little instruction in the duties which they will be called on to fulfil in after life, determined to establish schools for the teaching of domestic economy. The idea is not original, and domestic economy classes are to be found in our own primary schools, but the methods followed in Belgium are so much more practical than our own, that it may be worth while to give some account of them. The weak point of English teaching of domestic economy is that it is not sufficiently practical. Girls learn cooking with a gas-stove by which they can regulate the heat to a nicety, and which needs no stoking; they are taught to wash clothes which are already clean; in sewing they produce garments elaborately tucked and feather-stiched—much too dainty for daily use; and even their mending is taught on holes of mathematical accuracy—and nobody tears or wears their clothes mathematically. Thus, while our teaching certainly gives some notion of women's work, it cannot be said to turn out girls capable of meeting the emergencies of ordinary life in humble circumstances.

The Belgian authorities have gone on the principles of teaching their pupil to use the materials and means which lie to their hand. In the cookery classes the girls are first taught marketing—a thing wholly omitted in our system. The younger girls are accompanied by a mistress when they go out to buy the materials for the meal that is to be the subject of the lesson, but the older girls are allowed to go alone to do the purchasing. Only where markets are a long way off does the mistress provide the ingredients, and then she makes an imaginary sale of them to the pupils. It may be asked wherein lies the educational value of sending a child to buy half a pound of sugar or two ounces of butter instead of doling out to her these commodities from a cupboard. If the teaching was only a matter of instruction in the preparing of certain dishes according to the rules of a cookery book, there would be none. But the problem set before the Belgian girl is a different one. She is asked to make out the menu of the dinner as well as to cook it. The dinner is to be sufficient for six persons—that being taken as the average number in a family—and the cost is not to exceed a franc and a half—that is to say, about twopence-halfpenny a head. For this sum the girls are expected to put on the table a soup, and a meat course with a vegetable. In districts where the rate of wages is high it is permitted, in view of the comparative luxury possible in the children's homes, to add a third course, the cost of which must not exceed half a franc, or rather less than a penny a head. To produce, under these restrictions, a meal which shall meet the necessary requirements, demands intelligent purchasing as well as skilful cooking, and it is in this and not in the absolute teaching of cookery that our system fails. Another point may be noted—that when the meal has been prepared the girls sit down and eat it in the presence of their teacher. In some schools the girls take it in turns to carve the meat and serve the vegetables. "It is found," says the report of these schools, "that sitting down to table in an orderly fashion very rapidly leads to a marked improvement in the manners of the girls. Its usefulness may be inferred from the fact that girls sometimes enter these classes ignorant of the use of a fork." The apparatus used for these cooking lessons is of the utmost simplicity. A stove with a small oven, worth about £2, such as is found in most working-men's cottages, is used, and the fuel is invariably wood or coal.

Laundry work also is taught with the most simple materials. Each school has one or two ordinary round wooden wash-tubs. "Occasionally a rude kind of washing machine, worth a few francs, is used, but mangles seem to be unknown." The children are taught all the various processes of laundry work, from soaking to starching and ironing, and the garments on which they practise are brought from their own homes. The same is the case with the sewing. General methods of sewing are taught in the junior classes, but a girl who has entered the domestic economy class is supposed to know the various stitches, and is allowed to do her work under only the general supervision of the mistress. Mending is regarded as of more importance than making in the course of instruction. An English visitor to these schools notes that the children bring from home "stockings, tablecloths, and towels to be darned, children's pinafores to be patched, men's trousers to be reseated, dresses that the older children have outgrown to be cut down and refashioned for the little ones." As for the way in which these tasks are performed, it is admitted that "the results as regards stitching are by no means such as would satisfy the inspectress in an English school, accustomed to the work of specimens; but the work as a whole quite reaches the level that could fairly be expected from the busy mother of a large family; the darns will last certainly as long as the garments." This is surely more important than the producing of "specimens." Even when whole garments are made attention is devoted to the production of many useful things—strong dresses for the pupils' own wear, and shirts and blouses for their fathers and brothers—than to the elaboration of one. Advanced pupils are taught cutting-out by means either of patterns or measurements, and they are sometimes allowed to use a sewing-machine. In addition, the girls are taught to clean stuff garments with benzine, ammonia, and the like, and to take out grease spots in various ways.

The cleaning of the school is done by these domestic economy pupils. They sweep and scrub the floors, clean the windows and the paint, and occasionally give the furniture a coat of varnish. In some schools the girls bring boots, knives, spoons and forks, copper and tin saucepans from their homes, and are taught at school the method and materials needed for the cleaning of each. Lamp-trimming is another subject of study.

The theoretic part of household management is not neglected. Lessons are given upon the cost and arrangement of furniture, the arrangement of house work for a day and a week, with special instructions regarding spring cleaning. A little about nursing is taught, and the pupils are told of household remedies for cuts, burns and bruises. Still it is in the practical teaching of ordinary household work that these Belgian schools excel. The result of the teaching has been, from an official point of view, only too successful The schools were begun with a view to the training of potential wives, but it is found that clever girls who have profited by the teaching are not always inclined to settle down to humble domestic life, but are able to find good situations as dressmakers, ladies' maids, etc. It is doubtful, however, if there is anything to grumble at in women being made competent and independent in any sphere, and no doubt the majority of the pupils marry in the long run, and their husbands, to whatever class they belong, are the gainers by the thorough instruction their wives have received.

